# What does standard rehabilitation practice after total hip replacement in the UK entail? results of a mixed methods study

**DOI:** 10.1186/1471-2474-14-91

**Published:** 2013-03-12

**Authors:** Tosan Okoro, Ashok Ramavath, Jan Howarth, Jane Jenkinson, Peter Maddison, John G Andrew, Andrew Lemmey

**Affiliations:** 1 School of Medical Sciences, Bangor University, Bangor, UK; 2 School of Sports Health and Exercise Science, Bangor University, Bangor, UK; 3 Department of Orthopaedics, Ysbyty Gwynedd, Betsi Cadwaladr University Health Board, Bangor, UK; 4 Department of Physiotherapy, Ysbyty Gwynedd, Betsi Cadwaladr University Health Board, Bangor, UK

**Keywords:** Physiotherapy, Orthopaedics, Osteoarthritis, Training

## Abstract

**Background:**

There is evidence of prolonged poor function in patients following total hip replacement (THR). Studies of progressive resistance training (PRT) interventions to improve function are often compared to ‘standard’ practice which is not well defined. This study aimed to investigate ‘standard’ rehabilitation care in the UK after total hip replacement (THR) as well as determine whether PRT was part of ‘standard’ care.

**Methods:**

After ethical approval, questionnaire item development about rehabilitation practice was guided by a focus group interview (after informed consent) with physiotherapists (n = 4; >5 years post-qualification) who regularly treated THR patients. An online questionnaire investigating the exercises prescribed and rehabilitation practice following THR was developed and sent to physiotherapists working in hospitals in the UK. The survey was performed from January to May 2011. The survey results were analysed (frequency (%) of responses) focusing on the exercises the physiotherapists considered important, as well as their use of PRT in prescribed regimes.

**Results:**

106 responses were obtained from physiotherapists in the UK. The survey respondents considered that the most important muscles to target in all phases of rehabilitation were the hip abductors (62.2%), followed by the quadriceps (16.9%), and other muscles (21%). Exercise type prescribed revealed no consensus, with weight bearing (42%), functional (45%) and Bed-based/Bridging/Postural exercises (13%) favoured. 83.7% were able to define the basis of progressive resistance training (PRT), but only 33% prescribed it.

**Conclusions:**

Standard physiotherapy rehabilitation in the UK after THR is variable, and appears to rarely include PRT. This may be a factor in prolonged poor function in some patients after this common operation.

## Background

Total hip replacement (THR) is one of the most widely performed procedures in orthopaedic practice and with the increasing age of the population; the number of persons who require such surgery is on the rise, [1]. In England and Wales, according to the National joint registry, the number of primary total hip replacements (THR) in 2009/2010 totalled 79413, which is a steady rise from the amount reported in 2008/2009 (77608) and 2007/2008 (73632), [2]. THR has revolutionalised the care of patients with end stage joint disease, leading to pain relief, and substantial improvement in quality of life, [3]. However, long-term studies show that, even in the absence of pain, there is persistence of impairment and functional limitation, [4]. These long lasting impairments include reduced muscle strength, reduced postural stability, and limited flexibility, [5]. The functional limitations include reduced walking speed, and lower ratings on various assessment tools used to measure functional ability compared with those who have never had hip arthritis, [5].

Considerable technical efforts have been made towards optimising THR; for example, there are over 100 varieties of hip prostheses, multiple bearing couples, and several surgical approaches. However, the actual health gain for many of these innovations is small in terms of patient function and quality of life, [6]. In tandem with these technical developments, patient expectations, including for an early return to normal physical function and activities; have also increased, [7]. In the recent past, a prolonged hospital stay after THR incorporated a period of rehabilitation. However, due to the introduction of initiatives such as integrated care pathways and considerations of cost and patient satisfaction, the length of hospital stay following joint replacement has reduced markedly over the past decade from a mean of 3 weeks to 4 days [8].

Since the evidence suggests patients who score poorly on subjectively assessed functional outcome measures 2 years post-operatively after THR are five times more likely to require assistance with ADLs compared to those who have good function, [9], rehabilitation may therefore increasingly important to avoid long-term impairment and to optimise functional recovery, [5].

Prior to surgery, there is a general deficit in muscular strength along the affected limb as compared to the contra-lateral (healthy) side in patients with unilateral hip osteoarthritis (OA), and muscles such as the abductors, vastii, rectus femoris and psoas show marked atrophy, [10]. Immobilisation due to major surgery and hospitalisation can cause a further decline in muscle mass, muscle strength and muscle function, [11]. Muscle strength declines 4% per day during the first week of immobilisation, and the deficits in the involved hip after THR range from 10-21% when compared to the uninvolved hip at 1 year post-surgery, [12,13] the atrophic changes that occur about the hip persist up to 2 years following THR, [10].

The most commonly used rehabilitation regimes for older people are based on functional types of exercises which do not involve external loading and have not been shown to prevent further muscle atrophy after THR [5,14]. In contrast, progressive resistance training (PRT) is an effective method for inducing muscle hypertrophy and increasing muscle strength and functional performance in healthy and clinical populations, including the elderly, [15]. PRT elicits positive health and performance adaptations by challenging the skeletal muscles with loads that can be lifted repetitively until the onset of neuromuscular fatigue, i.e. the point at which appropriate technique can no longer be maintained, [15]. To facilitate continued adaptation, training load is progressively increased, and exercises are adjusted as indicated throughout the training regimen, to attenuate the onset of a plateau in physiological adaptation.

PRT in rehabilitation following THR has been shown to significantly enhance muscle strength and function, [16-18] with PRT being the main factor in achieving significant functional improvements in rehabilitation regimes used after home or centre based regimes used after THR, [19]. Although a plethora of studies exist testing different rehabilitation protocols (including PRT) against ‘standard’ practice, [20,21] no explicit definition is made as to what ‘standard practice’ entails. Clarification of the variation in the available rehabilitation programs after this common operation is therefore important. The aims of this study were firstly to define standard rehabilitation care following THR in the UK and secondarily to determine whether PRT is prescribed as part of standard care.

## Methods

After local NHS Research Ethics Committee approval, a focus group interview was convened. A heterogenous sample of physiotherapy participants were purposively sampled using the following criteria: 1) a minimum of 5 years experience post-qualification, 2) experience of treating patients following THR and 3) working in either the inpatient, outpatient and community environment with patients following THR. The participants were accessed through physiotherapy management leads that provided a suitable list and the above criterion were used to approach potential candidates. Of the four physiotherapists subsequently recruited, two worked in the hospital setting, one worked in the community and the other was outpatient based.

After informed consent, the focus group was conducted by the first author (TO; main moderator) with a co-author (AR) acting as a co-moderator and scribe. A number of topic areas were discussed with the group with the questions posed based on a preliminary review of the literature from which a number of gaps appeared [20,21].

The focus group lasted a total of 90 minutes. The results of the meeting were transcribed (summary provided in Table 1) with content analysis and theming [22,23] performed in order to develop questionnaire domains and items.

**Table 1:** Summary of focus group findings assessing suitable issues in investigating rehabilitation practice after total hip replacement in the UK

**Discussion item**	**Answers**
*What do you do with your hip replacements?*	In bed exercises
	Quadriceps static exercises
	Free range abductors
	Bridging exercises
	Hydrotherapy
*Could you tell us a little bit about the exercise/physical activity you prescribe generically for your hip replacement patients?*	Depends on period of rehabilitation
	For Mobility: strengthening, functional, balance and weight bearing
	Active instructions given are determined by time/service pressures
	Target quadriceps and glutei muscles
*What exercises do you think are important for total hip replacement surgery patients?*	In bed exercises
	Quadriceps static
	Free range abductors
	Bridging exercises
	Hydrotherapy
	Strengthening
	Functional
	Balance
	Weight bearing
*What would you like to know about a colleague’s practice with regards total hip replacement rehabilitation?*	Demographics of patients seen?
	Who mobilises first following surgery?
	Day patient mobilised post-operatively?
	Average duration of inpatient of stay?
	Grade of physiotherapist that sees patients
	Pre-operative education provided?
	Use of resistance training?
	**Inpatient rehabilitation**
	Grade of physiotherapist that sees patients
	Equipments patients are discharged with
	**Outpatient rehabilitation**
	Intermediate care team visits at home?
	Mechanism/ route of re-referral?
	Inpatient and outpatient discharge criteria?
	Number of times patients seen in outpatient clinic
	Frequency of visits and number seen personally

These items reflected rehabilitation practice in the pre-operative, immediate post-operative and continuing rehabilitation phases, as defined by the physiotherapy focus group (Table 2). The questionnaire was piloted with five local physiotherapists and no further changes were made before incorporation of the questions into an online questionnaire hosted by a secure survey system (Bristol online survey: URL http://www.survey.bris.ac.uk). A total of 171 hospitals were contacted (128 from England, 27 from Scotland, 10 in Wales and 7 from Northern Ireland) with a total of 63 email addresses (England n = 41, Scotland n = 12, Wales n = 8, Northern Ireland n = 2) obtained from physiotherapists directly involved in THR care. An online internet link was also posted on the Chartered Society of Physiotherapists website (URL: http://www.csp.org.uk). The online survey was run from January to May 2011. It was not possible to estimate a response rate as the number of possible viewings on the CSP website could not be estimated and responses from emails obtained were anonymised. The questionnaire was also tailored to collect demographic information as to the Physiotherapists’ grade, the type of centre they worked in (NHS or private), and the average number of THR patients managed post operatively per year. The survey results were analysed focusing on the exercises the physiotherapists considered most important, as well as the understanding and use of PRT in prescribed rehabilitation regimes. The questions posed were analysed by assessing the percentage of respondents from the total sample.

**Table 2:** Questionnaire items on rehabilitation practice for total hip replacement

**Rehabilitation Phase**	**Question posed with possible responses**
Preoperative	In what setting do you see patients in the preoperative period?
	*Response options:* Orthopaedic Pre-assessment clinic
	Outpatients
	Arthroplasty education event
	Other- Please state…….
	Which exercise do you personally think is most important in terms of muscular recovery after total hip arthroplasty?
	*Response options:* Weight-bearing exercises
	In-bed exercises
	Bridging exercises
	Functional exercises
	Hydrotherapy
	Which muscle group do you think it is most important to target following total hip arthroplasty for rehabilitation purposes?
	*Response options:* Quadriceps
	Hip abductors
	Spinal
	Core balance
	Other-please state
	What kind of advice do you give?
	*Response options:* Verbal
	Trust information leaflet/booklet
	Video/CD
	Other…..
Immediate	Which healthcare professional mobilises the patient out of bed first?
Post-operative	*Response options:* Nurses
Immediate Post-operative	Physiotherapists
	Other- Please state…………
	On average what day do the patients get mobilised?
	*Response options:* Day 0
	Post op day 1
	Post op day 2
	Post op day 3
	What exercises do you prescribe to your patients?
	*Response options:* Weight bearing exercises
	In-bed exercises
	Bridging exercises
	Functional exercises
	Hydrotherapy
	Other- Please state
	Which one of the following options do you think most underlines progressive resistance training?
	*Response options:* Progression of frequency of exercises
	Use of weights
	Do you build in resistance training routinely into the exercises you prescribe?
	*Response options:* Yes
	No
	Which of the following is the average length of stay in your hospital after total hip arthroplasty?
	*Response options:* 2 days
	3 days
	4 days
	>5 days
	How often is a post-operative hip patient seen routinely by you in a normal day? *Response options:* Once
	Twice
	Thrice
	Four times
	Other
	What equipments are your patients routinely discharged from hospital with?
	*Response options:* Walking aids
	Thromboprophylaxis e.g. Clexane
	Other- Please state……………..
	By what criteria do you discharge your patients from inpatient physiotherapy input?
	*Response options:* Patient stable on walking aid
	Compliant with exercises
	Independent with activities of daily living (ADL)
	Other- Please state………………
	Do you routinely refer patients to community physiotherapy on discharge?
	*Response options:* Yes
	No
Continuing rehabilitation	What is the reason for referral of patients to your care in the weeks or months after THR in the community?
Continuing rehabilitation	*Response options:* Poor balance
	Weak hip abductors
	Frequent falls
	Recurrent dislocation
	Other- Please state…
	What exercises do you prescribe for patients after THR in the community?
	*Response options:* Weight bearing exercises
	In-bed exercises
	Bridging exercises
	Functional exercises
	Hydrotherapy
	Other- Please state
	By what criteria do you discharge them from your care?
	*Response options:* Patient stable on walking aid
	Compliant with exercises
	Other- Please state………………
	How many sessions do you on average see the patient in total?
	*Response options:* 1
	2
	3
	4
	5
	>5

## Results

106 responses were received from physiotherapists who supervise rehabilitation of THR patients in the UK. Of these, 85% worked in the NHS and 15% in the private sector. 104 out of 106 physiotherapists (98.1%) who responded were Band 5 and above (average of 5 years post-qualification experience), and 94% of all respondents worked in centres performing greater than 100 THR operations per year, Table 3. The responses to the questions posed in each of the rehabilitation phases are detailed below:

**Table 3:** Comparison of physiotherapy respondents based on the number of total hip replacement operations performed per year in their centres of work

**Number of operations per year**	**Number of responses**	**Frequency (%)**
<100	7	6.6
100-200	18	17
200-300	35	33
>300	46	43.4
Total	106	100

### Preoperative phase

The majority of the respondents (67.9%) saw patients in the preoperative period; mainly in the pre-assessment clinic (36.1%) or arthroplasty education seminars (33.3%), with general outpatients (8.3%), inpatient ward review and home visits (22.2%) making up the remainder. The advice given preoperatively consisted of a trust booklet (39%), verbal advice (7%), a video/CD (2%), or a combination of these (25%). The hip abductors were considered to be the most important muscle group to target during rehabilitation (62.2%), followed by the quadriceps (16.9%) and others such as the hip flexors, extensors, spinal, and core muscles (21%), Table 4. No consensus existed as to which form of exercise was most important in the initial phases of rehabilitation; with weight bearing (performed against gravity, 42%) and functional (without external loading, 45%) being the most favoured, and 13% of responses indicating a preference for bed-based (e.g. buttock squeezes, leg sliding and straight leg raise)/bridging (targeting core abdominal muscles as well as lower back and hip)/postural exercises (focusing on strengthening muscles which have become overstretched and weak).

**Table 4:** Muscle groups felt to be most important to target by physiotherapists’ surveyed in the UK with regards to rehabilitation regimes after total hip replacement surgery

	**Actual responses obtained with frequencies (%)**				
**Band of Physiotherapist**	**Quadriceps**	**Hip Abductors**	**Spinal/Core Muscles**	**Other**	**Totals**
2	0	0	0	0	0
3	1	0	0	0	1
4	0	0	1	0	1
5	3	6	1	0	10
6	5	15	0	7	27
7	9	35	1	5	50
8	0	10	1	6	17
Totals	18 (16.9%)	66 (62.2%)	4 (3.7%)	18 (16.9%)	106 (100%)

### Immediate postoperative phase

The length of stay after THR was reported to range between 1 and >5 days with a stay of 4 days eliciting the most frequent response (57.5%). 86.6% (n = 92) of the physiotherapists who responded to the survey tend to mobilise the patients themselves on day 1 with the remainder mobilised by physiotherapy assistants and nurses. Patients were reviewed at least once a day 36.6% of the time (n = 37) with twice daily reviews performed by a majority of physiotherapists surveyed (52.5% n = 53). 85% of all respondents used a combination of weight bearing, bed, and functional exercises. 83.7% of respondents knew what PRT entailed, but only 33% routinely included it in the exercises prescribed following THR, Table 5. Patients were discharged based on a combination of being compliant with exercises, stable with a walking aid, and independence with activities of daily living (Table 6), with all patients provided with walking aids and 71% of respondents suggesting that patients went home from their institutions with thromboprophylaxis. 74.5% of the survey respondents did not routinely refer patients to community physiotherapy on their discharge from hospital.

**Table 5:** Responses of physiotherapists surveyed regarding prescription of progressive resistance training in standard rehabilitation after total hip replacement surgery

	**Questionnaire Item: Do you build in resistance training routinely into the exercises you prescribe;**		
	**Actual response (frequency (%))**		
Band of Physiotherapist	Yes	No	Totals
2	0	0	0
3	0	1	1
4	0	1	1
5	2	8	10
6	9	18	27
7	19	31	50
8	5	12	17
Totals	35 (33%)	71 (66.7%)	106

**Table 6:** Discharge criteria and review frequency for patients reviewed in the immediate post-operative and continuing rehabilitation phases after total hip replacement surgery according to physiotherapists surveyed in the UK

**Phase of rehabilitation**	**Discharge criteria (% of responses)**	**Number of times patients seen (% of responses)**
**Immediate post-operative** (n = 106 physiotherapists)		
	Stable on walking aid (85.8%)	*In a day:*
	Compliant with exercises (57.2%)	Once (36.6%)
	Independent with activities of daily living (66%)	Twice (52.5%)
		Thrice (2%)
**Continuing rehabilitation** (n = 48 respondents involved in this phase)		
	Stable on walking aid (33.3%)	*Total number of sessions*
	Compliant with exercises (25%)	*patients seen in total:*
	Independent with activities of daily living (41.7%)	Once (24.7%)
		Twice (13.5%)
		Thrice (20.2%)
		Four times (20.2%)
		Five times (9%)
		>Five times (12.4%)

### Continuing rehabilitation

A total of 48 (45.3%) respondents saw patients on an outpatient basis weeks/months after discharge whilst 58 (54.7%) respondents did not. 48% of the physiotherapists involved in this phase of rehabilitation thought weak hip abductors were the most common reason for referral as opposed to poor balance (34%), recurrent dislocation (13%) and frequent falls (5%). The exercises prescribed during this period consisted of functional (62.1%), weight bearing (17.7%) and bed exercises (6.9%), with the use of PRT not evident in the responses obtained. The discharge criteria and number of times patients were reviewed in this phase of rehabilitation are described in Table 6.

## Discussion

Despite 83.7% of the survey respondents being able to ascertain what PRT entailed, only 33% routinely built it into their prescribed rehabilitation programs. 48% of the survey respondents who treated THR patients in the outpatient setting reported that weak hip abductors accounted for the majority of referrals in the ‘continuing rehabilitation’ phase, with poor balance accounting for 34%, and prescriptions given were mainly of functional, weight bearing and bed exercises. The reasons for referral reflect a lack of muscle strength which is consistent with the established findings after THR as already described [10]. The use of PRT therefore helps address this deficit and prevent further muscle atrophy.

This is the first study in the published literature that attempts to quantify the standard practice for physiotherapy rehabilitation following THR surgery in the UK. Recent systematic reviews have looked at programmes and interventions to improve functional outcome in this group of patients, [19-21] and a common observation made is the variety in the ‘standard’ or control regimes that are used for comparison. With the inpatient stay reduced to an average of 4 days as reported by a majority of the physiotherapists surveyed in this study, intervening following patient discharge from hospital is increasingly important if the persisting functional deficits typical of these patients are to be resolved.

In the preoperative phase of rehabilitation, 67.9% of the U.K. physiotherapists surveyed had contact with patients in a variety of settings. Preoperative counselling and education reduces unrealistic expectations regarding pain as well as improves patient satisfaction, [24]. Preoperative physiotherapy on the other hand reportedly improves muscle strength and gait, allowing early return to ambulatory function, [20] but its impact on short-term outcome such as gait speed, cadence, walking distance is debatable as the trials performed also include post-operative interventions on the same cohort of patients, [20]. The uses of the techniques described (arthroplasty education seminar, trust booklets, etc.) are therefore advantageous and exercise prescription in this phase of rehabilitation did not emerge as a theme from the convened focus group.

Due to the current financial pressures the NHS is under with staffing levels and patient throughput, [25], the primary objective for the majority of physiotherapists may be to mobilise the patients for discharge. Criteria used include stability with a walking aid, compliance with the prescribed exercises, and independence with ADLs. Inherently therefore the objective is to aim for safety on mobilisation and functional optimisation is not top of the agenda. Possible issues that may hinder physiotherapists in using PRT include time constraints as already described with possibly referral patterns, difficulties in transporting patients to a gym setting, and the demand on inpatient services meaning that the primary objective for the majority of working physiotherapists is to aim for safety on mobilisation. As intervening to improve function is increasingly difficult to commence in the hospital setting due to the pressures to achieve a short inpatient stay, referral to community physiotherapy to continue appropriate regimes may be what is required. There are inherent difficulties with this approach as 74.5% of the survey respondents did not routinely do this, illustrating the difficulties of adapting practice to reflect the significant evidence base. It is also possible that the closed nature of the question posed in the questionnaire meant that some physiotherapists who may include an element of PRT occasionally in the regimes prescribed responded negatively. Patients may not be routinely referred to outpatient physiotherapy post-operatively perhaps due to different local protocols and guidelines with care funding packages. It was also evident from the focus group discussion that routine outpatient assessment post-operatively was not considered except patients were anticipated to have problems with mobilization.

The orthopaedic surgeons’ role in the rehabilitation recommendations is mainly determined by the integrity of the implants used. Recent literature suggests that early full weight bearing with cementless implants may have no significant impact on primary stability and bony ingrowth [26] making exercise prescription by physiotherapists in the early post-operative phase more relevant.

From the 7^th^ annual report of the NJR in 2010, [2], 392 centres entered data on the numbers of THR surgery performed up to the year end in 2009. Of the 106 survey respondents, 100 (94%) worked in centres performing >100 THR procedures per year (217/392 centres according to the NJR data) perhaps implying that about half of the hospitals listed were surveyed. A more comprehensive survey of these centres may have provided more generalizable information but this was inherently difficult as only 63 emails addresses were obtained from 171 hospitals contacted (36.8%). The remainder of responses obtained can be assumed to be from a combination of colleagues of those in whom emails were sent as well as physiotherapists with access to the CSP website.

The use of the focus group to determine the questionnaire items for the survey further validates the findings obtained. This survey aimed to determine ‘standard’ practice and the 4 physiotherapists used in the focus group were from a mixture of backgrounds but all treated THR patients regularly. Since no questionnaire existed to achieve the aim of this study, the focus group ensured that the items obtained helped clarify any underlying assumptions of personal or traditional practice such as frequency of times patients need to be seen, exercises that should be prescribed, etc. Sources of information and experience as well perspectives and viewpoints of the professional group to be studied are also better integrated using this method of qualitative research [27]. The focus group participants in this instance were more likely to feel a sense of community and belonging which makes them more comfortable and more willing to express opinions [27], therefore ensuring that the items obtained for the questionnaire used reflect the pre-operative, post-operative and continuing rehabilitation phases as already described.

A limitation of this study is the fact that no analysis of regional variations in practice was performed. This was in part due to an effort to anonymise the data obtained so as to preserve the confidentiality of respondents as required by the ethics committee. An effort was also made not to identify areas which may have huge disparity in service provision from the norm. Further information may also have been gleaned from content analysis of the preoperative advice and instructions given to patients in the form of booklets, videos and CDs. Such an undertaking was not feasible in this instance but perhaps can be part of a future study to further clarify what standard THR rehabilitation regimes entail.

## Conclusions

THR surgery is an increasingly common operation for hip osteoarthritis. This survey demonstrates that physiotherapy practice in the UK rarely includes progressive resistance exercise; and this may be a factor in prolonged poor function in some patients. Future research should focus on the issues restricting implementation of suitable PRT regimes to improve functional outcome.

## Competing interests

The authors declare that they have no competing interests.

## Authors’ contributions

TO was responsible for study design, facilitation of the focus group, administration of the survey, interpretation of the study results and manuscript preparation. AR, JH and JJ were involved in convening the focus group as well as interpreting the data collected and administration of the questionnaire. JGA conceived the study and in conjunction with PM and AL, was involved in overall supervision of the study, and review of manuscript drafts. All authors read and approved the final manuscript.

## Pre-publication history

The pre-publication history for this paper can be accessed here:

http://www.biomedcentral.com/1471-2474/14/91/prepub
